# FABP4, GINS2 and CBX7 Expression in Cancer Cervix Tissues: Clinical, Pathological and Prognostic Implications

**DOI:** 10.30699/IJP.2023.1971325.2944

**Published:** 2023-12-29

**Authors:** Mariem A Elfeky, Rema H Faraj Saad, Mohamed Ali Alabiad, Mohammed Alorini, Rehab Hemeda, Ramadan M Ali, Loay M. Gertallah, Mohamed Negm, Ahmed Mahmoud Abdou, Ahmed Baker A Alshaikh, Ahmed Elmaasrawy

**Affiliations:** 1 *Department of Pathology, Zagazig University Faculty of Medicine, Zagazig, Egypt*; 2 *Department of Pathology, Faculty of Medicine, University of Benghazi, Benghazi, Libya*; 3 *Department of Basic Medical Sciences, Unaizah College of Medicine and Medical Sciences, Qassim University, Unaizah, Kingdom of Saudi Arabia*; 4 *Department of Clinical Oncology and Nuclear Medicine, Faculty of Medicine, Zagazig, Egypt*; 5 *Department of General Surgery, Faculty of Medicine, Zagazig University Zagazig, Egypt *; 6 *Department of Gynecology and Obstetrics, Zagazig University Faculty of Medicine, Zagazig, Egypt*; 7 *Department of Obstetrics and Gynecology, College of Medicine, Jouf University, Sakaka, Kingdom of Saudi Arabia*

**Keywords:** FABP4, GINS2, CBX7, Cancer cervix, Immunohistochemistry, Prognosis

## Abstract

**Background & Objective:**

Cervical cancer spreads to the pelvic lymph nodes, leading to a high incidence of cancer recurrence and unfavorable survival rates. Therefore, there is an urgent need to detect new predictive biomarkers for the early assessment of pelvic lymph node status in patients with cervical cancer. The current study aimed to assess the expression of FABP4, GINS2, and CBX7 in cervical cancer tissue to detect their prognostic and predictive roles in developing lymph node metastases in patients with that cancer type.

**Methods:**

We collected the tissues from patients with cervical cancer and evaluated the expression of FABP4, GINS2, and CBX7 using immunohistochemistry. We evaluated the association between their expression and clinicopathological and prognostic parameters.

**Results:**

A high expression of FABP4 and GINS2 and a low expression of CBX7 were found to be positively associated with the old age group, large tumor size, high grade and lymphovascular involvement, para-uterine organ infiltration, advanced FIGO stage, chemotherapeutic resistance, and tumor recurrence.

**Conclusion:**

We demonstrated the oncogenic roles of FABP4 and GISN2 in addition to the on-co-suppressive roles of CBX7 in cervical cancer and their association with poor clinicopathological criteria and poor survival. Our results may indicate that FABP4, GISN2, and CBX7 could be considered predictive biomarkers of the occurrence of lymph node metastases in the cancer of the cervix preoperatively, which could be beneficial in the accurate preoperative design therapy.

## Introduction

The cancer of cervix is ranked as the second most common cancer and a common cause of cancer-related death, particularly in developing countries (1). Cervical cancer spreads to pelvic lymph nodes, leading to a high incidence of cancer recurrence and unfavorable survival rates (2, 3). Advancements in surgical management and chemoradiotherapy could not improve patient outcomes, particularly if pelvic lymph node metastases were found (4). The European Society of Gynecological Oncology guidelines recommend a preoperative evaluation of pelvic lymph nodes for early design of management strategies (5). Therefore, it is urgent to detect new predictive biomarkers for the early assessment of pelvic lymph node status in patients with cervical cancer (6, 7)**.**

Fatty acid binding protein 4 (FABP4) is an intracellular lipid chaperone that can carry fatty acids to many organelles (8). Recent studies have incriminated FABP4 in carcinogenesis and metastatic potential of cancers of many organs such as colon, breast, and ovary (9).

Subunit 2 of the GINS complex (GINS2), which belongs to the GINS complex family, encodes a protein that plays a role in initiating DNA replication and controlling cell cycle and normal cell division. GINS2 expression was found in many cancer types and was associated with carcinogenesis, cancer progression, and metastases (10). Epithelial–mesenchymal transition (EMT) is characterized by a change of malignant cells from epithelial to spindle cells that can invade and metastasize. Chromobox homologue 7 (CBX7) was found to promote cancer occurrence and spread through the promotion of EMT. CBX7 was found to have several functions in cancer according to cancer type (11). 

However, the prognostic and predictive functions of FABP4, GINS2, and CBX7 in the cancer cervix and their association with the occurrence of lymph node metastases are not sufficiently clarified.

The current study aimed to assess the expression of FABP4, GINS2, and CBX7 in cancer cervix tissue to detect their prognostic and predictive roles in developing lymph node metastases in cancer cervix patients.

## Material and Methods

We included 62 patients with cancer cervix who underwent a radical hysterectomy in addition to lymphadenectomy in the Gynecology and Obstetrics Department and General Surgery Department, Faculty of Medicine, Zagazig University, from 2017 to 2022. The patients were followed to detect disease recurrence and survival rates. Tissue samples were sent to the Pathology Department, Faculty of Medicine, Zagazig University, where they were processed, diagnosed, graded, and staged. Sections from paraffin blocks of prepared samples are stained with FABP4, GINS2, and CBX7 using immunohistochemistry.

**Inclusion Criteria**

Patients with a sure diagnosis of operable cervical carcinoma, Stages I-III, who accepted to be included in the study.

**Exclusion Criteria**

Inoperable patients are patients who received preoperative chemotherapy or radiotherapy.

**Ethical Approval:** The local ethics committee of the Faculty of Medicine of Zagazig University approved the study with an approval code of (ZU-IRB #10140). Written informed consent was obtained from all patients prior to enrolment in the study. 

**Immunohistochemistry:**

Sections from paraffin blocks of included cases were incubated with rabbit monoclonal anti-FABP4 antibody (1:50 dilution; Proteintech, 12802- 1-AP, China), anti-GINS2 antibody (1:600; HPA057285; Sigma) and anti-CBX7 (1:200 dilution; ab2187 (12-17)3; Abcam, Cambridge, UK). Markers expression was evaluated by assessment of intensity and extent of the stain: staining intensity (no = 0, weak = 1, moderate = 2, strong = 3) and extent (no = 0, less than 30% = 1, between 30 and 60% = 2, more than 60% = 3). The final stain index was reached by multiplication of the stain intensity and extent scores, reaching values of 0-9. A stain score ≥4 indicates high expression; a score of <4 indicates low expression.

**Statistical Analysis**

We performed statistical analyses using Graph Pad software (version 7.0). FABP4, GINS2, and CBX7 expression levels and association with clinicopathological and prognostic parameters were analyzed by the χ2 test. We measured DFS and OS rates using the Kaplan–Meier method and analyzed the differences in survival using the Log-rank test. The univariate and multivariate Cox proportional hazard models tested the predictive values of these ten variables. All statistical tests were two-sided. A P-value< 0.05 was considered statistically significant.

## Results

**Fig. 1:**
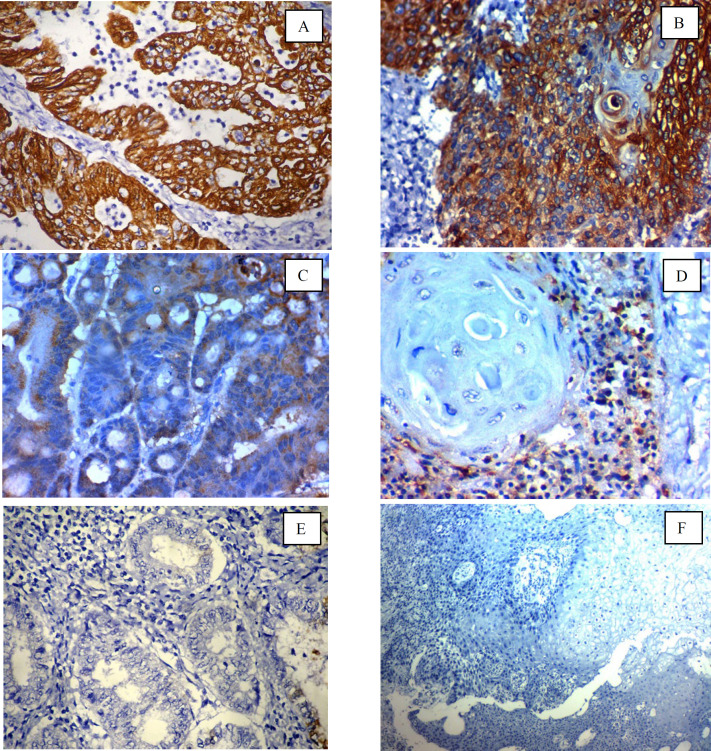
Immunohistochemical expression of FABP4 in cervical carcinoma: (A); high cytoplasmic expression in high-grade adeno-carcinoma of the cervix ×400, (B); high cytoplasmic expression in high-grade squamous cell carcinoma of the cervix (C); Low cytoplasmic expression in low-grade adeno-carcinoma of the cervix ×400, (D); Low cytoplasmic expression in low-grade squamous cell carcinoma of the cervix (E) ); Negative cytoplasmic expression in low-grade adeno-carcinoma of the cervix ×400, (F) Negative cytoplasmic expression in low-grade squamous cell carcinoma of the cervix x 200

The demographic and clinicopathological parameters of the included patients are summarized in Table 1. The expression of FABP4 expression was upregulated in cervical cancer tissues. Its expression was positively associated with the age of the patient (*P*=0.002), the size of the large tumor (*P*=0.004), the high grade (*P*=0.005), involvement of the lymphatic vascular space, infiltration of para-uterine organs, advanced stage of FIGO, resistance to chemotherapy and tumor recurrence (*P*<0.001). Patients with high expression of FABP4 had shorter rates of recurrence-free survival (RFS) and overall survival (OS) rates (*P*<0.001) Figures 1 and 4; Tables 2, 3, 4, 5, and 6.

The expression of GINS2 expression was increased in cervical cancer tissues. Its expression was positively associated with the old age of the patient (*P*=0.002), large tumor size (*P*=0.004), high grade (*P*=0.005), involvement of the lymphovascular space, infiltration of para-uterine organs, advanced stage of FIGO, resistance to chemotherapy, tumor recurrence (*P*<0.001).

Patients with high expression of GINS2 had shorter rates of recurrence-free survival (RFS) and overall survival (OS) rates (*P*<0.001) Figures 1 and 4; Tables 2, 3, 4, 5, and 6.

The expression of CBX7 expression was markedly downregulated in cervical cancer tissues. High expression of CBX7 was associated with a low grade (*P*=0.049), absence of myometrial invasion (*P*=0.039), absence of lymphovascular space involvement, absence of para-uterine infiltration, early stage of FIGO (*P*=0.022), response to chemotherapy (*P*=0.33) and lower incidence of tumor recurrence (*P*=0.028).

Patients with high CBX7 expression had longer recurrence-free survival (RFS) and overall survival (OS) rates (*P*<0.001) Figures 3 and 4; Tables 2, 3, 4, 5, and 6.

**Fig. 2:**
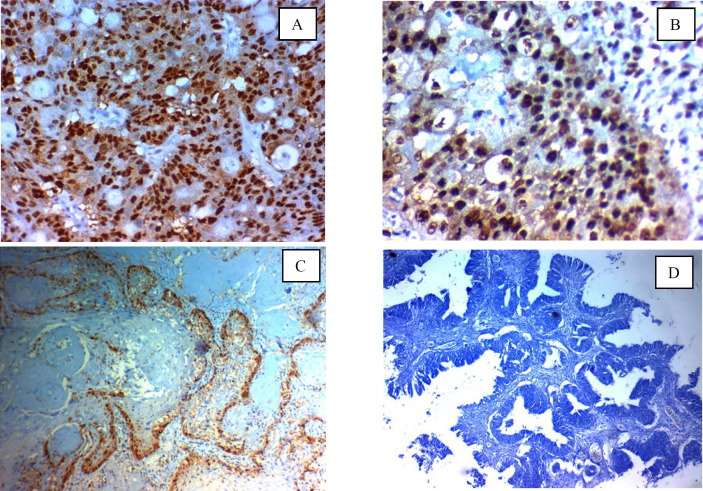
Immunohistochemical expression of GINS2 in cervical carcinoma: (A); high nuclear expression in high grade adenocarcinoma of the cervix ×400, (B); high nuclear expression in high grade squamous cell carcinoma of the cervix (C); Low nuclear expression in low grade squamous cell carcinoma of the cervix ×400, (D); Negative nuclear expression in low grade adeno-carcinoma of the cervix x 200

**Fig. 3:**
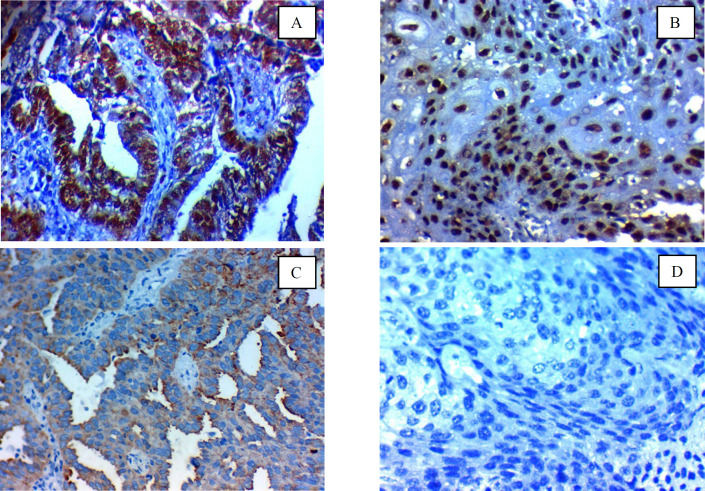
Immunohistochemical expression of CBX7 in cervical carcinoma: (A); high nuclear expression in low-grade adenocarcinoma of the cervix ×400, (B); high nuclear expression in low-grade squamous cell carcinoma of the cervix (C); Low nuclear expression in high-grade adenocarcinoma of the cervix ×400, (D); Negative nuclear expression in high-grade squamous cell carcinoma of the cervix x 400

**Fig. 4:**
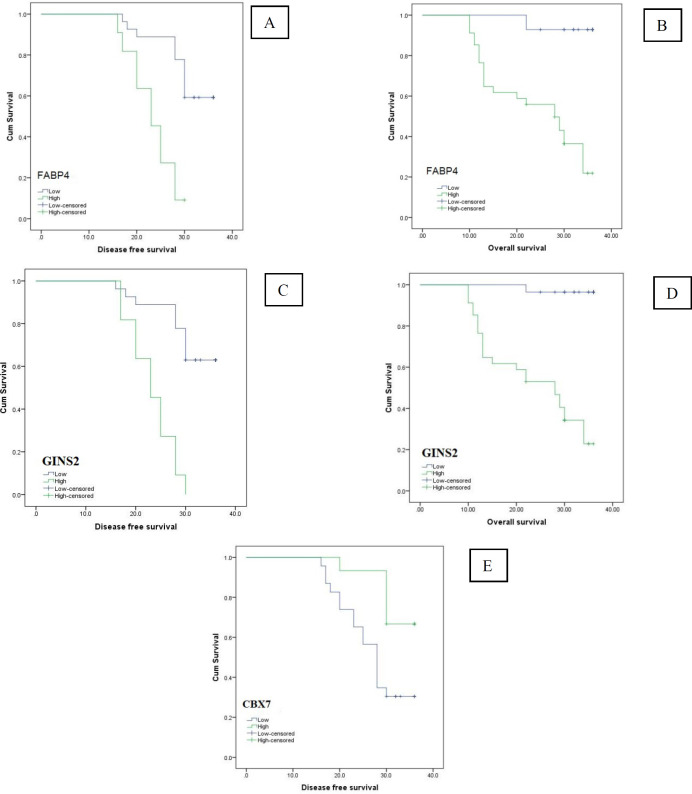
Kaplan Meir survival curves of DFS and OS rates of the studied cervical carcinoma patients: (A, C, E) DFS rates of the studied cervical carcinoma cases stratified according to FABP4, **GINS2** and CBX7 expression respectively, (B, D, F) OS rates of the studied cervical carcinoma cases stratified according to FABP4, **GINS2** and CBX7 expression, respectively

NUSAP1: nucleolar and spindle associated protein 1; MELK: maternal embryonic leucine zipper kinase; L1CAM: L1 cell adhesion molecule; OS: overall survival; DFS: disease-free survival

**Table 1:** Relationship between FABP4, GINS2, and CBX7 levels in the studied patients and their demographic and disease-specific characteristics

			FABP4			GINS2			CBX7		
			Low	High	P^¥^	Low	High	P^¥^	Low	High	P^¥^
	N=62	%	N=28 (45.2%)	N=34 (54.8%)		N=28 (45.2%)	N=34 (54.8%)		N=40 (61.3%)	N=22 (38.7%)	
Age group:											
≤ 55 years old	25	40.3	26 (92.7)	11 (32.4)	<0.001*	16 (57.1)	9 (26.5)	0.014*	9 (22.5)	16 (72.7)	<0.001*
>55 years old	37	59.7	2 (7.1)	23 (67.6)		12 (42.9)	25 (73.5)		31 (77.5)	6 (27.3)	
Histopatholog:											
Squamous cell carcinoma	44	71	19 (67.9)	25 (73.5)	0.78	19 (67.9)	25 (73.5)	0.78	28 (70)	16 (72.7)	0.821
Adenocarcinoma	18	29	9 (32.1)	9 (26.5)		9 (32.1)	9 (26.5)		12 (30)	6 (27.3)	
Size:											
<4cm	6	9.7	6 (21.4)	0 (0)	0.006*	6 (21.4)	0 (0)	0.006*	3 (7.5)	3 (13.6)	0.657
≥4cm	56	90.3	22(78.6)	34 (100)		22 (78.6)	34 (100)		37 (92.5)	24 (86.4)	
Grade:											
I	10	16.1	10 (35.7)	0 (0)	<0.001*	10 (35.7)	0 (0)	<0.001*	4 (10)	6 (27.3)	0.303
II	38	61.3	17 (60.7)	21 (61.8)		16 (57.1)	22 (64.7)		27 (67.5)	11 (50)	
III	14	22.6	1 (3.6)	13 (38.2)		2 (7.1)	12 (35.3)		9 (22.5)	5 (22.7)	
LVSI:											
Absent	44	71	27 (96.4)	17 (50)	<0.001*	26 (92.9)	18 (52.9)	0.001*	28 (70)	16 (72.7)	0.821
Present	18	29	1 (3.6)	17 (50)		2 (7.1)	16 (47.1)		12 (30)	6 (27.3)	
Lymph node:											
Absent	30	48.4	22 (78.6)	8 (23.5)	<0.001*	22 (78.6)	8 (23.5)	0.002*	17 (42.5)	13 (59.1)	0.211
Present	32	51.6	6 (21.4)	26 (76.5)		6 (21.4)	26 (76.5)		23 (57.5)	9 (40.9)	
Distant metastasis:											
Absent	46	74.2	27 (96.4)	19 (55.9)	<0.001*	20 (71.4)	10 (29.4)	0.003*	29 (72.5)	17 (77.3)	0.681
Present	16	25.8	1 (3.6)	15 (44.1)		8 (28.6)	24 (70.6)		11 (27.5)	5 (27.7)	
Stage:											
I	6	9.7	6 (21.4)	0 (0)	<0.001*	6 (21.4)	0 (0)	0.001*	3 (7.5)	3 (13.6)	0.286
II	24	38.7	16 (57.1)	8 (23.5)		14 (50)	10 (29.4)		14 (35)	10 (45.5)	
III	16	25.8	5 (17.9)	11 (32.4)		6 (21.4)	10 (29.4)		12 (30)	4 (18.2)	
IV	16	25.8	1 (3.6)	15 (44.1)		2 (7.1)	14 (41.2)		11 (27.5)	5 (22.7)	

*P<0.05 is statistically significant ^¥ ^Chi square test

**Table 2:** Distribution of the studied patients according to treatment-specific characteristics and patients’ outcomes

	N = 62	%
Treatment:		
Surgery Surgery and radiotherapy Surgery and chemotherapy Surgery, radiotherapy**,** and chemotherapy Radiotherapy Chemotherapy	13 10 17 14 4 4	21 16.1 27.4 22.6 6.5 6.5
Response:		
PD SD PR CR	37 4 7 14	59.7 6.5 11.3 22.6
Response:		
OAR NR	41 21	66.1 33.9
Outcome:		
Alive Dead	37 25	59.7 40.3
Disease free survival (months) (N=38):		
Mean ± SD Range	29.03 ± 6.49 16 - 36	
Overall survival (months):		
Mean ± SD Range	27.68 ± 9.18 10 – 36	

**Table 3:** Relationship between FABP4, GINS2, and CBX7 levels in the studied patients and treatment-specific characteristics and patients’ outcomes

	FABP4			GINS2			CBX7		
	Low	High	*P*	Low	High	*P*	Low	High	*P*
	N=28(45.2%)	N=34(54.8%)		N=28(45.2%)	N=34 (54.8%)		N=40 (64.5%)	N=22((35.5%)	
Treatment response:									
CR	27 (96.4)	10 (29.4)	<0.001*	26 (92.9)	11(32.4)	<0.001*	32 (57.5)	14 (63.6)	0.933
PR	1 (3.6)	3 (8.8)		2 (7.1)	2 (5.9)		3 (7.5)	1 (4.5)	
SD	0 (0)	7 (20.6)		0 (0)	7 (20.6)		4 (10)	3 (13.6)	
PD	0 (0)	14 (41.2)		0 (0)	14(41.2)		10 (25)	4 (18.2)	
Response:									
OAR	28 (100)	13(38.2)	<0.001*	28 (100)	13(38.2)	<0.001*	26 (65)	15(68.2)	0.8
NR	0 (0)	25(61.8)		0 (0)	21(61.8)		14 (35)	7 (31.8)	
Recurrence (n=38):									
Absent	16(59.3)	1 (9.1)	<0.001*	17 (63)	0 (0)	<0.001*	7 (30.4)	10(66.7)	0.028*
Present	11(40.7)	10(90.9)		10 (37)	11 (100)		16(69.6)	5 (33.3)	

*P<0.05 is statistically significant ¥ Chi square test

**Table 4:** Relationship between FABP4, GINS2, and CBX7 levels in the studied patients and treatment-specific characteristics and patients’ outcomes

	FABP4			GINS2			CBX7		
	Low	High	*P*	Low	High	*P*	Low	High	*P*
	N=28 (45.2%)	N=34 (54.8%)		N=28 (45.2%)	N=34 (54.8%)		N=38 (61.3%)	N=24 (38.7%)	
Treatment response:									
CR	27 (96.4)	10 (29.4)	<0.001*	26 (92.9)	11 (32.4)	<0.001*	31 (81.6)	6 (25)	<0.001*
PR	1 (3.6)	3 (8.8)		2 (7.1)	2 (5.9)		4 (10.5)	0 (0)	
SD	0 (0)	7 (20.6)		0 (0)	7 (20.6)		2 (5.3)	5 (20.8)	
PD	0 (0)	14 (41.2)		0 (0)	14 (41.2)		1 (2.6)	13 (54.2)	
Response:									
OAR	28 (100)	13 (38.2)	<0.001*	28 (100	13 (38.2)	<0.001*	35 (92.1)	6 (25)	<0.001*
NR	0 (0)	25 (61.8)		0 (0)	21 (61.8)		3 (7.9)	18 (75)	
Recurrence (n=38):									
Absent	16 (59.3)	1 (9.1)	<0.001*	17 (63)	0 (0)	<0.001*	17 (53.1)	0 (0)	<0.001*
Present	11 (40.7)	10 (90.9)		10 (37)	11 (100)		15 (46.9)	6 (100)	
Disease free survival:									
Mean ± SD	31.41±5.62	23.18 ± 4.58	<0.001*^∞^	33.69 ± 3.4	29.36±5.7	<0.001*^∞^	32.88±4.6	31.33±6.81	0.003*^∞^
Range	17 - 36	16 - 30		22 - 36	16 - 36		20 - 36	30 - 34	
Overall survival:									
Median	36	28	<0.001*^#^	32	23	<0.001*^#^	30	25	<0.001*^#^
Range	22 - 36	10 - 36		16 - 36	17 - 30		16 - 36	23 - 28	

t Independent sample t test ^∞^Z Mann-Whitney test *P<0.05 is statistically significant ^¥ ^Chi square test ^#^independent sample t test

**Table 5:** Correlation between FABP4, GINS2, CBX7 markers among the studied patients

	FABP4		GINS2		CBX7	
	**Phi**	***P***	**Phi**	***P***	**Phi**	***P***
FABP4			0.87	<0.001*	0.721	<0.00*
GINS2	0.87	<0.001*			0.721	<0.001*
CBX7	0.721	<0.001*	0.721	<0.001*		

**P≤0.001 is statistically highly significant

**Table 6:** Relationship between FABP4, GINS2, and CBX7 levels in the studied patients and their outcome

Patients’ outcome	FABP4		GINS2		CBX7	
	High	Low	High	Low	High	Low
	N=34 (%)	N=28 (%)	N=34 (%)	N=28 (%)	N=22 (%)	N=40 (%)
Outcome: Dead Alive	23 (67.6) 11 (32.4)	2 (7.1) 26 (92.9)	24 (70.6) 10 (29.4)	1 (3.6) 27 (96.4)	18 (81.8) 4 (18.2)	19 (47.5) 21 (52.5)
*P*	<0.001**		<0.001**		<0.008*	
Odds ratio	27.18		64.8		4.97	
95% confidence interval	5.45 – 136.68		7.72 – 544.14		1.43 – 17.34	

OR odds ratio CI confidence interval **P≤0.001 is statistically highly significant.

## Discussion

The current study showed that high FABP4 expression in cervical cancer tissues is associated with unfavorable prognostic parameters and poor outcomes. Furthermore, we showed that increased expression of FABP4 is associated with a higher incidence of lymph node metastases. Similarly, Guoqing Li *et al.* (6) showed that FABP4 is associated with the development of lymph node metastases and a poor prognosis in patients with cervical cancer. Our results are important because lymph node status is essential in preparing and assigning treatment in patients with cancer cervix. 

Furthermore, Guoqing Li *et al.* (6) showed that loss of FABP4 expression inhibits cervical cancer cell proliferation, invasion, and metastases. Taken together, FABP4 increased the malignant potential of cervical cancer cells. Thus, it could be a significant prognostic and predictive biomarker of lymph node metastases in cancer cervix patients (6).

It was recently found that there are many predictive risk factors for lymph node metastases in the cancer cervix, such as the advanced FIGO stage, the large size of the tumor, the depth of cervical stromal invasion, and lymph-vascular invasion (18-20). 

We showed that FABP4 was an essential predictive risk factor for lymph node metastases in cancer cervix patients, like Guoqing Li *et al.* (6), who hypothesized that the preoperative evaluation of FABP4 expression levels in colposcopy biopsy tissues could provide clinicians with key clues for better surgical and chemotherapeutic management.

Furthermore, we demonstrated a positive association between FABP4 expression and a poor OS rate; therefore, FABP4 might be a beneficial predictive and prognostic biomarker for cancer patients with cancer cervix. In addition to other studies, our findings indicate that more aggressive and individualized management strategies are needed to manage patients with FABP4 overexpression (6).

The occurrence of lymph node metastases is a complex process controlled by many genes that increase the invasiveness of cancer cells (21). According to Guoqing Li *et al.* (6), FABP4 has a role in controlling epithelial markers, and its over-expression could increase mesenchymal markers, facilitating the metastatic process. Moreover, it was observed that specific inhibitors of FABP4 were found to decrease cancer progression by reducing proliferation, invasive ability, and metastasis of cancer cells. Furthermore, FABP4 enhances cancer progression by controlling lipid metabolism, the AKT pathway, and stimulating EMT (22). 

According to Zhang *et al.*, the expression of FABP4 in CRC tissues is positively associated with cancer progression and lymph node metastases (23). Luo *et al. *(24) revealed a similar role in patients with pancreatic adenocarcinoma tissues.

In contrast to our results, Zhong *et al.* (25) reported an inverse association between FABP4 expression levels, proliferative and invasive liability of hepatocellular carcinoma cells, and its low expression levels related to unfavorable survival rates (25). Different roles in different types of cancer may explain these results. Therefore, more studies are needed to clarify the precision of FABP4 in the cancer cervix.

We evaluated another novel expression of prognostic markers that has not been markedly evaluated in cervical cancer, GINS2. We showed that GINS2 is upregulated in cervical cancer tissues, associated with well-established poor prognostic parameters such as high-grade deep stromal invasion, and positively correlated with poor survival and unfavorable outcomes. Our results were in line with Fei Ouyang *et al.* (7), who reported a high expression of GINS2 in cervical cancer tissues and an association with unfavorable outcomes. Moreover, they showed that down-regulation of GINS2 down-regulation reduces cancer cells, proliferation, invasion, and metastases; thus, it has a role in the progression of cancer cervix.

A similar prognostic role of GINS2 up-regulation is present in other cancers, such as lung, breast, and cholangiocarcinoma (26, 27). Furthermore, high expression of GINS2 was related to cancer stem cell activation and progression in breast cancer (10). Our results indicated that GINS2 plays many roles in cancer oncogenesis and progression. 

Many mechanisms explain the prognostic roles of GINS2 in cancer, such as cancer stem cell activation, inhibition of apoptosis, and cell cycle control. In cancer cervix, evaluation of the status of lymph nodes is needed preoperatively for an accurate designation of treatment strategy (28). Performing lymphadenectomy in early-stage patients without lymph node metastases leads to morbidity and postoperative complications (29). There is no accurate predictive biomarker or imaging technique for preoperative diagnosis of lymph node metastases; also, sentinel lymph node biopsy could not accurately assess its occurrence (30, 31). 

According to our findings, GINS2 overexpression was positively correlated with lymph node metastases and aggressive cancer cervix; therefore, it could be considered a novel therapeutic target and prognostic marker. 

We showed that both FABP4 and GISN2 are upregulated in cancer cervix tissues and are related to unfavorable outcomes.

We evaluated the expression of another biomarker (CBX7) in cervical cancer tissues to confirm the role of these novel biomarkers.

Our results indicated that CBX7 negative expression was associated with poor prognostic parameters and unfavorable outcomes in cervical cancer patients. Similarly, the results of Ping Tian* et al.* and Ramirez *et al.* (11, 32) showed that the absence of CBX7 in cancer cervix tissues was related to unfavorable prognostic parameters, such as the presence of lymph nodes and blood metastases, to unfavorable survival outcomes. 

Our results were in line with previous results in other cancers, such as glioma and pancreatic carcinoma (33, 34).

Ping Tian *et al.* (11) explained the oncogenic functions of loss of CBX7 by reducing levels of E-cadherin in tumor cells, thus increasing invasion and metastases by losing epithelial characteristics and having a mesenchymal phenotype that promotes the progression of different types of cancers by the initiation of EMT (35, 36). Unlike our results, previous studies by Federico *et al.* (37), Sepe *et al.* (38), and Shinjo *et al.* (39) showed that CBX7 might play an oncogenic role in cancer progression in some cancers, such as ovarian and colon cancer.

We showed a positive association between FABP4 and GISN2 expression levels in cancer cervix tissues, and both were inversely associated with the expression of CBX7.

## Conclusion

We reported prognostic roles of FABP4, GISN2, and CBX7 in cervical cancer tissues, and we demonstrated oncogenic roles of FABP4 and GISN2 in addition to onco-suppressive roles of CBX7 in cervical cancer. Our results indicated that FABP4, GISN2, and CBX7 might be considered predictive biomarkers to the occurrence of lymph node metastases in the cancer cervix preoperatively, which could be beneficial in accurate preoperative designing therapy.

We evaluated the expression of novel biomarkers not extensively studied in cervical cancer. Furthermore, we assessed the expression of these markers in different grades and stages of cancer cervix patients. 

Although our research highlighted the important aim of gynecologic surgeons by evaluating novel predictive biomarkers of lymph node metastases in cancer cervix preoperatively, there are some limitations, such as small sample size and using only immunohistochemistry for tissue protein expression of the biomarkers. To validate our findings, we recommend including a larger cohort of patients with cervical cancer patients; additionally, a molecular evaluation of the markers is needed to prove our findings.

## Conflict of Interest

None.
